# The d16HER2 Splice Variant: A Friend or Foe of HER2-Positive Cancers?

**DOI:** 10.3390/cancers11070902

**Published:** 2019-06-28

**Authors:** Lorenzo Castagnoli, Michael Ladomery, Elda Tagliabue, Serenella M. Pupa

**Affiliations:** 1 Molecular Targeting Unit, Department of Research, Fondazione IRCCS Istituto Nazionale dei Tumori, 20133 Milan, Italy; 2 Faculty of Health and Applied Sciences, University of the West of England, Coldharbour Lane, Frenchay, Bristol BS16 1QY, UK

**Keywords:** d16HER2 splice variant, wild-type HER2, breast cancer, regulation of alternative splicing, tumor aggressiveness, cancer stem cells, targeted therapy

## Abstract

Human epidermal growth factor receptor 2 (*ERBB2* or HER2) amplification/overexpression is associated with a particularly aggressive molecular subtype of breast cancer (BC), characterized by a poor prognosis, increased metastatic potential, and disease recurrence. As only approximately 50% of HER2-positive patients respond to HER2-targeted therapies, greater knowledge of the biology of HER2 and the mechanisms that underlie drug susceptibility is needed to improve cure rates. Evidence suggests that the coexistence of full-length, wild-type HER2 (wtHER2) and altered forms of HER2—such as carboxy-terminus-truncated fragments, activating mutations, and splice variants—significantly increases the heterogeneity of HER2-positive disease, affecting its biology, clinical course, and treatment response. In particular, expression of the d16HER2 splice variant in human HER2-positive BC has a crucial pathobiological function, wherein the absence of sixteen amino acids from the extracellular domain induces the formation of stable and constitutively active HER2 homodimers on the tumor cell surface. Notably, the d16HER2 variant significantly influences the initiation and aggressiveness of tumors, cancer stem cell properties, epithelial–mesenchymal transition (EMT), and the susceptibility of HER2-positive BC cells to trastuzumab compared with its wtHER2 counterpart, thus constituting a novel and potentially clinically useful biomarker. The aims of this review are to summarize the existing evidence regarding the pathobiological functions of the d16HER2 variant and discuss its current and future value with regard to risk assessment and treatment choices in HER2-positive disease.

## 1. Introduction

Since its identification in 1985 [1], the human *ERBB2* (HER2) oncogene has ranked highest with regard to its relevance in oncology, especially breast cancer (BC). This gene encodes the 185-kD transmembrane human epidermal growth factor receptor 2 (HER2), which belongs to the HER family of receptors—including its relatives, HER1 (or EGFR), HER3, and HER4 [2]. These proteins consist of an extracellular domain (ECD) that binds growth factors, a transmembrane lipophilic segment, and an intracellular tyrosine kinase domain [3]. Activation of their tyrosine kinase domain generally occurs through homodimerization and heterodimerization that are induced by a specific ligand [3]. 

Once activated, cell signaling through HER family receptors leads to proliferation and survival [4]. HER2 is an exception to the canonical activation mechanism, because it lacks specific growth factor ligands and becomes activated due to its fixed conformation, which resembles a ligand-activated condition [5]. This property is why HER2 is the preferred heterodimerization partner for other HER receptors [2].

The HER2 receptor is amplified or overexpressed in 15% to 20% of invasive BCs, and is associated with more aggressive disease and worse outcomes [6]. Increased levels of HER2 in overexpressing BC versus normal breast tissue, its function in tumor aggressiveness, and its surface expression on tumor cells make HER2 an ideal molecule against which targeted therapies can be developed [4]. The advent of HER2-targeted agents, particularly trastuzumab, a recombinant humanized monoclonal antibody that is directed towards HER2, has revolutionized the treatment of this aggressive BC subtype, significantly improving overall survival in advanced and primary BC patients. Although the collective results suggest a clinical benefit for trastuzumab, this antibody, administered per currently approved protocols, only eradicates the disease in approximately 50% of patients with HER2-positive early BC and cannot cure those with HER2-positive metastatic tumors (reviewed in [7]) .

The complexity of the HER2 proteome is well known, and various isoforms, generated through several mechanisms—such as proteolytic cleavage, alternative initiation of translation, somatic mutations, and alternative pre-mRNA splicing—have been described (Table 1). There are at least three well-established splice variants of HER2 [8]. Herstatin is a truncated version of HER2, with 79 additional amino acids at the C-terminus that are encoded by retained intron 8 [9]. The p100 HER2 isoform is also a truncated version of HER2 and arises from the retention of intron 15 [10]. These truncated versions inhibit cell proliferation by interfering with HER2 dimerization and activation and, consequently, the growth of HER2-positive tumor cells [11].

The third reported variant, which we have named d16HER2—also referred to as ΔHER2, HER2Δ16, Δ16HER2, delta16HER2, Delta-HER2, ErbB2DeltaEx16, and Delta16—has the opposite functions of herstatin and p100. d16HER2 is derived from the skipping of exon 16, which encodes a small region of HER2/ECD [12]. Understanding the function of HER2 splice variants—specifically of d16HER2—might be crucial in understanding the biology and aggressiveness of HER2-positive BCs and their response to targeted therapies. 

Here, we summarize the studies that have described the generation of d16HER2 and its involvement in BC, focusing on HER2-driven tumorigenesis, tumor aggressiveness, stemness, epithelial–mesenchymal transition (EMT) programs, and the response to anti-HER2 biodrugs. Establishing the function of d16HER2 in the progression of HER2-positive BC is needed to define its clinical value and determine whether it should be considered in the selection of therapeutic approaches.

### 1.1. Alternative Splicing and the Regulation of d16HER2 Expression

Soon after the discovery of pre-mRNA splicing in the late 1970s, it became apparent that exons are not always spliced together in the same manner. Multi-exon genes can express several mRNAs using various combinations of exons. These mRNAs can encode ‘splice isoforms’ of proteins with disparate and even antagonistic properties.

Following this discovery, it was clear that alternative splicing is ubiquitous, resulting in an explosion of research on alternative splicing over the next decades. In humans, over 94% of multi-exon genes are believed to be alternatively spliced [13]. Many reviews have been written on the importance of alternative splicing in cancer, including BC (for example, see recent reviews [14,15,16]. These reviews draw attention to the function of splice isoforms in oncogenic processes and suggest that targeting the machinery of alternative splicing constitutes a novel therapeutic approach. 

Alternative splicing is regulated by splice factors, which are generally RNA-binding proteins that recognize specific sequences or RNA structures in exons and introns. However, it can also be controlled by noncoding RNAs. Splice factors bind to specific regulatory sequences, known as exonic or intronic splice enhancers or silencers (ESEs, ESSs, ISEs, and ISSs). These splice factors can then block or facilitate the recognition of splice sites by the spliceosome machinery. 

Splice factor activity is dysregulated in cancer. SRSF1 was the first splice factor to be described as a proto-oncogene in BC [17]. SRSF1 is a member of the SR (serine/arginine-rich) family of splice factors. SR proteins typically contain an RNA recognition motif (RRM) and an SR domain that facilitates protein—protein interactions. 

The activity of splice factors such as SRSF1 is regulated through phosphorylation by splice factor kinases, including SRPKs (SR protein kinases) and CLKs (CDC2-like protein kinases). SRPK1 drives the metastasis of BC cells [18], and its expression is associated with the progression of BC and poor patient survival [19]. The phosphorylation of SRSF1 by SRPK1 upregulates proangiogenic splice isoforms of VEGF-A [20]. Further, the inhibition of SRPK1 drastically inhibits tumor growth in vivo by blocking angiogenesis [21]. Similarly, the inhibition of the splice factor kinase CLK2 blocks growth in an allograft model of MYC-driven BC [22].

The splice isoform d16HER2 arises from exon 16 being skipped. Exon 16 is 48 bases long, and its skipping causes an in-frame loss of 16 amino acids. d16HER2 is expressed in nearly all BC samples, ranging in proportion from 4% to 10% of full-length HER2, and in HER2-positive gastrointestinal cancers [23,24,25,26]. The biological and developmental functions, evolutionary origins, and conservation of d16HER2 remain poorly understood, as do the mechanisms that underpin the regulation of the skipping of exon 16. A recent study has provided initial insights into what might regulate both p100 and d16HER2 expression, implicating the splice factors SRSF3 and hnRNPH1. The knockdown of hnRNPH1 increases the expression of d16HER2, and that of SRSF3 results in a switch from d16HER2 to p100, correlating with the inhibition of cell proliferation [27]. The complete profile of the splice factors (and, potentially, splice factor kinases) that regulate the inclusion of exon 16 in HER2 remains to be determined.

**Figure 1:**
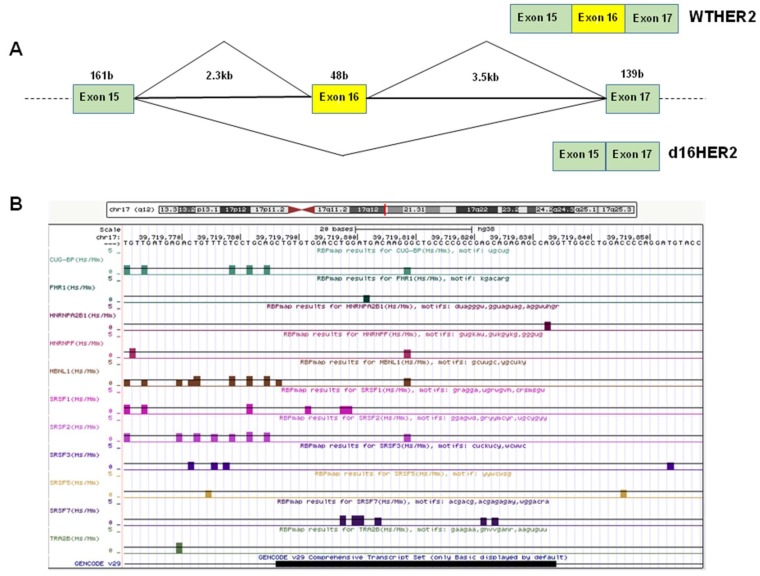
Analysis of *HER2*'s alternatively spliced cassette exon 16 and surrounding intron sequence. (**A**) Schematic of *HER2* splice isoforms (wtHER2, with exon 16 included and HER2, exon 16 skipped). (**B**) Analysis of human *HER2* exon 16 and flanking intronic sequence. The 3′ splice site, comprising the pyrimidine tract (underlined) is ctgtttctcctgcagCTG and the 5′ splice site is CTGgttggcctg. Putative binding sites are indicated for the splice factors CUG-BP, FMR1, hnRNPA2B1, hnRNPF, MBNL1, SRSF1, SRSF2, SRSF3, SRSF5, SRSF7, and TRA2B. The data was obtained using RBPmap (rbpmap.technion.ac.il) and visualised on the UCSC genome browser.

We have examined the sequence of exon 16 and its surrounding intronic sequences for the presence of splice factor binding sites (Figure 1) using the RBPmap algorithm [28]. We noted several potential binding sites for CUG-BP, FMR1, hnRNPA2B1, hnRNPF, MBNL1, SRSF1, SRSF2, SRSF3, SRSF5, SRSF7, and TRA2B, amongst others. Several of these splice factors have been implicated in BC. For example, MBNL1 has anti-metastatic properties, binding to the 3’ UTRs of mRNAs that encode suppressors of BC metastasis and stabilizing them [29]. TRA2B promotes the inclusion of cassette exons of *CD44* that are associated with BC metastasis [30]. The presence of potential SRSF1 binding sites is also notable, given the involvement of SRSF1 and of protein kinases that regulate SRSF1 activity in BC. 

#### d16HER2: A Crucial Driver of HER2-Driven Tumor Aggressiveness

In human primary BC, HER2 overexpression—due primarily to gene amplification—is relevant but insufficient to induce transformation [31]. In this context, the generation of ad hoc mouse models that are transgenic for rodent and human HER2 isoforms has allowed us to understand the molecular and genetic events that underlie the function of HER2 in the initiation and progression of BC and its response to targeted therapies [32].

In particular, to become oncogenic in vivo, the rodent HER2*/neu* and human HER2 transgenes require specific activating in-frame deletions or insertions of cysteine residues in the HER2/*neu* ECD that induce conformational changes in the juxtamembrane region of the receptor [32,33]. The resulting imbalance in cysteine residues is central to the regulation of the catalytic activity of HER2, causing activation of the receptor by promoting constitutive homodimerization through the formation of stable intermolecular disulfide bonds [12,32,34,35,36]. Various studies have reported the constitutive expression of an alternative splice isoform of human HER2 that carries an in-frame deletion in the same region that is mutated in the rat HER2*/neu* proto-oncogene in HER2-positive BC models [12,36]. This splice variant produces a dysfunctional HER2 receptor that lacks exon 16 and, in turn, 16 amino acids (634–649) in the HER2/ECD fragment, including two cysteine residues that are crucial to the assembly of HER2—exposing an unpaired cysteine that becomes available for intermolecular disulfide bonding. As shown in several engineered tumor cell models, this alteration induces the formation of constitutively active stable and covalently bound d16HER2 homodimers that have greater transformation activity than full-length/wtHER2 [12,23,24,37,38,39,40].

The first clinical evidence that coupled the expression of the d16HER2 variant with tumor progression and metastasis was reported in 2009 by Mitra and colleagues, who showed that 89% of patients with HER2-positive BC that coexpressed the d16HER2 variant presented with positive lymph nodes on diagnosis versus 12% of d16HER2-negative BCs [24]. The significant association of d16HER2 expression and positive lymph nodes (*p* < 0.0001) in their cohort implicated d16HER2 as a clinically important and tumor-specific molecular alteration of HER2 that promoted aggressive, locally disseminated metastatic BC. The authors claimed that the potentiated metastatic and oncogenic properties of d16HER2 were mediated through its direct coupling with Src kinase [24].

Then, d16HER2-driven oncogenic penetrance and its direct causative function in tumor onset and progression was examined by establishing a Friend Virus B-Type (FVB) transgenic mouse model for the human d16HER2 isoform. These mice develop spontaneous tumors, demonstrating that the d16HER2 isoform is perhaps the sole oncogenic driver of the human *ERBB2* proto-oncogene [25,37,40]. Such transgenic mice, termed the Δ16HER2-LUC line, develop several asynchronous metastatic mammary tumors between the age of 8 and 32 weeks, expressing heterogeneous levels of constitutively active, stable HER2 homodimers compared with the MMTVhuHER2 transgenic model, which form spontaneous human wtHER2-overexpressing mammary tumors after age 28 weeks [25,41].

Further, as shown in preclinical and clinical settings, compared with wtHER2, d16HER2 homodimers couple to several downstream oncogenic signal transduction pathways that increase the activation of Focal Adhesion Kinase (FAK), Phosphatidylinositol-3-Kinase/Protein Kinase B (PI3K/AKT), Mitogen-Activated Protein Kinase (MAPK) and Proto-oncogene tyrosine-protein kinase SRC (SRC), all of which have been implicated in tumor proliferation, migration, and induction of the EMT program [24,25,37,38]. In particular, the d16HER2-SRC axis, which amplifies d16HER2-driven oncogenic signals, is active in mammary adenocarcinomas that are transgenic for the human d16HER2 isoform [25,37], genetically engineered cell line models [24], and HER2-positive BC cases [25], confirming that activated SRC is the key surrogate marker that lies downstream of the oncogenic signalling of d16HER2. 

Consistent with these findings, Turpin et al. have shown that inducible expression of the d16HER2 variant, which they term ErbB2ΔEx16, in mammary glands of transgenic mice the rapid development of metastatic multifocal mammary tumors [39]. Unlike wtHER2-derived tumors, which express luminal keratins, ErbB2ΔEx16-derived tumors have distinct signaling and gene expression profiles that correlate with the activation of transcription factors that have been implicated in BC metastasis and cancer stem cell (CSC) renewal, such as activated Smad2, HIF1α, Stat3, and YB-1. 

Further, Alajati and colleagues used proteomic and genomic approaches in MCF10A cells that were engineered with a control vector, WT-HER2, or the d16HER2 isoform (Delta-HER2) [38]. By mass spectrometry, they identified a panel of known and novel phosphorylated signal transducers that lie downstream of HER2 in MCF10A-Delta-HER2 cells versus its counterparts. By microarray analysis of RNA that was extracted from the three cell lines, MCF10A-Delta-HER2 cells were enriched in genes that belonged to the growth factor/cytokine family and cell proliferation pathways. Notably, a search of a public BC dataset with Delta-HER2 signature genes showed that their specific enrichment correlated with ER-negative status, high tumor grade, and poor distant metastasis-free survival in metastatic BC patients [38]. 

### 1.2. d16HER2: The Chief Factor in HER2-Positive Breast Cancer Stem Cells and the EMT Program

Cancer stemness relies on the theoretical existence of a minor subset of cancer stem cells (CSCs) in the total tumor cell population that have the unique ability to self-renew and initiate a tumor [42]. In addition, several studies have shown that resistance to radiotherapy, chemotherapy, targeted therapy, and immunotherapy is attributable, at least in part, to CSCs, which are also linked to tumor metastasis [43,44]. 

Dynamic plasticity in breast CSCs (BCSCs) has been proposed to allow them to transit between EMT- and mesenchymal-epithelial transition (MET)-like states, endowing these cells with the capacity for tissue invasion, dissemination, and growth at metastatic sites [45]. Activation of the EMT state determines the loss of cell-to-cell contact, remodeling of the cytoskeleton, development of mesenchymal phenotypic traits [46], and acquisition of many CSC-associated properties by malignant cells [42]. Various signaling pathways that are dysregulated in CSCs, such as those that are driven by the NOTCH, HEDGEHOG, and WINGLESS (WNT) families, can also induce EMT [42].

In light of the important functions of CSCs in tumor biology, the response to therapy, and the prognosis, many studies have examined their biological properties and specific immunophenotype. In particular, altered expression and activity of the HER2 oncoprotein in HER2-positive BC is considered to be the master regulator of CSC/EMT programs [47]. Specifically, activity of the wtHER2 oncoprotein mediates the enrichment of the stem cell compartment in normal and malignant HER2-positive and -negative luminal mammary tumor cells [48,49]. In addition, we have explained the wtHER2 activity in the CSC compartment of HER2-positive BC, reporting novel functional crosstalk between wtHER2 and the NOTCH signaling pathway [50]. In parallel, wtHER2 overexpression correlates with the induction of the EMT state by downregulating E-cadherin and upmodulating mesenchymal markers, such as N-cadherin and Axl [51].

Recent evidence has implicated proteomic heterogeneity, determined primarily by alternative splicing of pre-mRNA, as one of the most significant mechanisms that contribute to the maintenance and differentiation of normal cells and CSCs [52,53] and induce the EMT program [54]. As discussed in the previous section, the d16HER2 variant is tumorigenic in vivo per se, with a shorter tumor latency compared with the wtHER2 form [37,41], and is critical for tumor aggressiveness, with significantly higher tumor penetrance versus the wtHER2 receptor [25]. 

The first evidence of the link between d16HER2 signaling and activation of the EMT program was provided by Mitra and colleagues [24], who showed that the d16HER2 and SRC signaling axis activated FAK, a key protein that is involved in BCSC activities and the EMT program [55]. Moreover, in luminal HER2-negative BC cell lines that ectopically expressed the human d16HER2 or wtHER2 isoform, based on the efficiency of colony formation—an in vitro assay that tests the stemness of cancer cell lines [56]—the number and size of d16HER2-positive colonies rose significantly versus wtHER2-positive colonies [24]. 

Alajati and colleagues compared the ability of d16HER2 and wtHER2 to sustain the EMT program, generating preliminary evidence that d16HER2 is critical in the regulation of the EMT program [38]. They observed that ectopic expression of d16HER2 in normal breast MCF10A cells simultaneously downregulated the epithelial markers cytokeratin 8 and E-cadherin and upregulated the mesenchymal marker N-cadherin, impaired the polarization of mammary acinar structures, and induced the invasion in 3D cell cultures. In contrast, transfection with the wtHER2 isoform failed to elicit a mesenchymal phenotype [38]. 

Further evidence of the ability of the d16HER2 and wtHER2 isoforms to drive EMT and enrich CSCs has been provided, based on the gene expression profiles of spontaneous mammary tumors that have been developed in transgenic mice that express human d16HER2 or wtHER2 [39]. These molecular analyses confirmed the selective upregulation of Smad-2 target genes, such as the TGF β superfamily, Snail1, and Twist1 [39] , all of which are believed to be critical for CSC activity and the EMT [57].

In murine adenocarcinoma cell lines that were transgenic for human d16HER2 or wtHER2 and ad hoc-engineered human MCF7 and T47D BC cell lines, the crucial function of the d16HER2 variant versus wtHER2 in HER2-positive BC stemness and aggressiveness has been demonstrated [58]. Moreover, using an activated d16HER2 metagene, obtained by comparing the gene expression profiles of human HER2-positive BCs according to d16HER2 and activated SRC expression levels (high versus low) [25], the authors found that expression and activation of the oncogenic d16HER2-pSRC signaling axis is the key driver of CSC enrichment in HER2-positive BCs, through its interaction with NOTCH family members and downstream signal transducers as HEY1, HES5, and HES6 molecules, whose significantly elevated levels expression are indicative of NOTCH pathway activation [58].

### 1.3. d16HER2: A Promising Predictor of HER2-Targeted Therapy

Based on its direct association with the ability to transform, compared with the wtHER2 and its significant enrichment in HER2-positive CSCs versus full-length HER2 receptor, the involvement of d16HER2 in conditioning the response to therapeutic approaches that target HER2 has been examined extensively in preclinical and clinical settings. In 2006, in HEK-293 cells that were transiently transfected with d16HER2, Castiglioni et al. showed that constitutive phosphorylation of disulfide bond-mediated homodimers was sensitive to Emodin, a tyrosine kinase inhibitor that affects the HER2 catalytic domain [23].

In contrast, the humanized antibody trastuzumab, which is the standard of care for patients with early and advanced HER2-positive BC, was found to bind d16HER2 to a lesser extent than full-length HER2 counterpart in HEK-293 transfectants, reflecting a lack of reactivity with d16HER2 homodimers that can be reverted in the presence of a reducing agent able to break disulfide bridges.

Because trastuzumab recognizes a conformational epitope in the juxtamembrane region of full-length HER2, in which sixteen amino acids are skipped in frame, we reasoned that this region is altered in d16HER2-transfected cells, impairing recognition by trastuzumab.

Based on these results, the involvement of d16HER2 in the antitumor activity of anti-HER2 reagents began to be studied. Consistent with our results [23], MCF-7 and NIH3T3 cell lines that express d16HER2 were found to be refractory to trastuzumab in vitro in cell proliferation and invasion bioassays, binding similarly to the d16HER2 and wtHER2 isoforms at the cell surface of MCF-7 transfectants [24]. Given the direct coupling of d16HER2 to SRC kinase in potentiating oncogenic properties, as discussed, dasatinib, a SRC tyrosine kinase inhibitor, suppressed tumorigenicity of this isoform in breast carcinoma cells [24].

Turpin and colleagues reported that d16HER2-expressing mammary tumor cell lines derived from ErbB2ΔEx16-transgenic mice or normal murine mammary gland (NmuMG) transfected with d16HER2 are resistant to the anti-HER2 therapeutic trastuzumab emtansine (T-DM1), an antibody–drug conjugate, whereas control cells that express wtHER2 are highly sensitive to it [39]. Given the very low internalization of d16HER2 reported in the same study, the authors speculated that the inability of T-DM1 to kill d16HER2-expressing cells depends on the inefficiency of the splice isoform to be internalized. Indeed, the delivery of T-DM1 requires internalization, and the consequent intracellular release of DM1-containing moieties through lysosomal degradation.

In contrast to in vitro studies that reported the resistance of d16HER2–expressing cells to the effects of trastuzumab, Alajati et al. showed that trastuzumab, administered in vivo to mice that had been xenografted with MCF10A cells that ectopically expressed d16HER2, blocked tumor growth [38], constituting the first evidence that the growth of tumor cells that are addicted to HER2 signaling can be blocked by anti-HER2 agents, even if the oncogene is primarily expressed as the d16HER2 splice isoform.

Consistent with the benefits of trastuzumab treatment observed in athymic mice xenografted with cells engineered to express d16HER2, we have found that the expression of d16HER2 improves the response to trastuzumab in d16HER2-positive transgenic mice [25]. Transgenic murine mammary cell lines that express the human d16HER2 variant, engrafted in syngeneic mice, become sensitive to lapatinib, a reversible inhibitor of the tyrosine kinase activity of HER1 and HER2 [59]. Moreover, using the “activated-d16HER2 metagene” to examine 2 datasets of HER2-positive BC patients who had been treated with trastuzumab-based neoadjuvant therapy or not, this metagene was more highly expressed in patients who achieved a complete or near-complete response to trastuzumab than in partial responders. Notably, no difference in the expression of the activated-d16HER2 metagene was seen between responders and nonresponders to neoadjuvant chemotherapy alone, supporting the benefit of trastuzumab in human BC with high d16HER2-dependent signaling [25]. Consistent with data in HER2-positive BC [23,25,58], we recently reported that d16HER2 is expressed in HER2-positive gastrointestinal cancers and that greater d16HER2 expression is associated with a clinical benefit and response to single-agent trastuzumab in patient-derived xenografts [26]. 

As full-length *ERBB2* and the d16HER2 splice variant are coexpressed in human HER2-positive BC, Palladini and colleagues studied their interaction and response to targeted therapy in hybrid transgenic mice that express full-length human *ERBB2* and d16HER2 (F1 HER2/Delta16), compared with parental HER2 and d16HER2 [40]. Trastuzumab inhibited tumor onset in F1 HER2/Delta16 and Delta16 mice but not in full-length HER2 mice, strengthening the concept that d16HER2 expression strongly contributes to tumor addiction to HER2 signaling and consequently susceptibility to HER2 blockade [40].

Tilio and colleagues [60] reported intrinsic resistance of d16HER2 transgenic females to lapatinib, wherein a slight delay in tumor onset and a minor reduction in tumor growth rate were induced by the treatment. In contrast, dacomitinib—an irreversible inhibitor of HER1 and HER2—inhibited the formation of autochthonous mammary tumors. The resistance to lapatinib was attributed to the preferential binding of this TKI to an inactive kinase conformation, whereas dacomitinib bound covalently and irreversibly to the active site of kinases. Saracatinib—a dual SRC/Abl kinase inhibitor—delays tumor onset and reduces tumor multiplicity in d16HER2 transgenic mice, confirming the involvement of SRC in d16HER2-driven carcinogenesis. Notably, tumors that develop in saracatinib-treated mice experience activation of the MAPK/Erk pathway, suggesting that this signaling compensates for the inhibition of d16HER2-dependent SRC activity.

In identifying microRNA suppressors of the oncogenic activity of d16HER2, Huynh et al. [61] found that the expression of d16HER2 in MCF-7 cells profoundly altered the expression of microRNAs, including the miR-7 tumor suppressor, and that the re-establishment of miR-7 expression sensitized refractory MCF-7/d16HER2 cells to trastuzumab [61]. 

In another study, it was reported that ectopic expression of d16HER2 in MCF-7 cells promotes tamoxifen resistance in vitro and in vivo through the upregulation of BCL-2 and the suppression of the BCL-2-targeting microRNAs miR-15a and miR-16. Reintroduction of miR-15a/16 downregulates tamoxifen-induced BCL-2 and sensitizes MCF-7/d16HER2 to tamoxifen. Conversely, inhibition of miR-15a/16 in tamoxifen-sensitive cells stimulates BCL-2 expression and promotes tamoxifen resistance [62]. Because ER is expressed in approximately 50% of HER2-positive BCs, Cittelly and colleagues have speculated that an expression analysis of d16HER2 and miR-15a/16 will yield better markers of tamoxifen resistance and novel targets for therapeutic intervention [62]. 

## 2. Conclusions 

**Figure 2:**
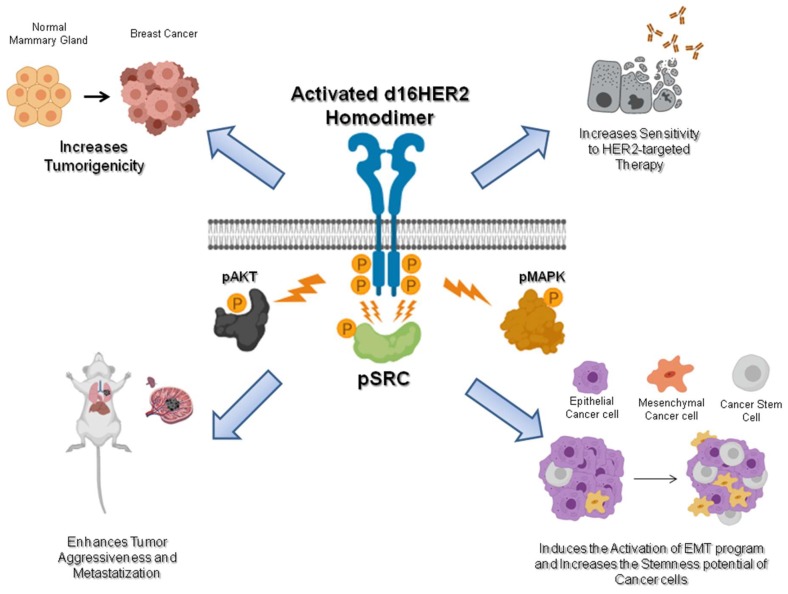
Schematic of the pathobiological features of d16HER2. The homodimerization of the d16HER2 splice isoform results in autophosphorylation of the tyrosine kinase domains and induction of downstream signaling. Cell signaling downstream of d16HER2 is transduced to the nucleus through different circuitries including Mitogen-Activated Protein Kinase (MAPK), Protein Kinase B (AKT), and mainly by Proto-oncogene tyrosine-protein kinase Src (SRC). Through the activation of these downstream pathways, d16HER2 is able to increase tumorigenicity and sensitivity to anti-HER2 drugs and enhance tumor aggressiveness and metastatization. d16HER2 also induces activation of the epithelial–mesenchymal transition (EMT) program and the enrichment of cancer stem cells (CSCs) inside the tumor. Created by Biorender.com.

In this review, we have discussed the growing body of evidence that the HER2 splice isoform d16HER2 has distinct and important pathobiological functions in HER2-positive BC. As summarized in Figure 2, a growing body of data indicates that d16HER2 mediates tumor initiation, proliferation, invasion, and stemness and optimal sensitivity to HER2-targeted therapy in HER2-positive BC. Notwithstanding the ability of human d16HER2 to induce spontaneous tumor onset in transgenic mice, the random appearance of the tumors likely reflects the need for additional genetic and epigenetic alterations to induce neoplastic transformation, necessitating further study to determine the oncogenic potential of d16HER2.

Furthermore, the efficacy of trastuzumab in d16HER2-overexpressing cancer models is controversial, given their resistance in vitro and sensitivity in vivo. This discrepancy might be due in part to the presence of host immunological machinery in vivo that is triggered by trastuzumab against HER2-overexpressing tumor cells. However, we cannot exclude the possibility that tumors that express high levels of d16HER2 are precisely those that express high levels of wtHER2 transcript, and are extremely addicted to HER2 signaling for growth and progression and thus sensitive to blockade of the oncoprotein [7,59]. Although the binding of trastuzumab to d16HER2 might be impeded by d16HER2 homodimers with disulfide bridges, the concomitant high expression of wtHER2 on the tumor cell membrane facilitates the binding and therapeutic activity of this biodrug. Overall, d16HER2 could be considered a marker of a tumor’s addiction to HER2, warranting clinical investigation as a marker of the susceptibility of HER2-driven cancers to trastuzumab. 

## Figures and Tables

**Table 1:** Biodiversity of the proteome encoded by full-length HER2.

Generation Mechanism	HER2 Isoforms	Cellular Localization
Proteolytic Cleavage	HER2-ECD (p110)	Soluble extracellular
	648-CTF	Anchored in cell membrane
Alternative Splicing	d16HER2	Transmembrane
	Herstatin	Soluble extracellular
	P100	Soluble extracellular
Alternative Initiation of translation	611-CTF (p95HER2)	Transmembrane
	687-CTF (p95cyto)	Soluble intracellular
Somatic Mutations	most missense mutations	20% HER2 extracellular domain
	duplications/insertions	80% HER2 transmembrane-extracellular domain
